# Neuroprotective Potential of Biflavone Ginkgetin: A Review

**DOI:** 10.3390/life13020562

**Published:** 2023-02-16

**Authors:** İ. İrem Tatlı Çankaya, Hari Prasad Devkota, Gokhan Zengin, Dunja Šamec

**Affiliations:** 1 Department of Pharmaceutical Botany, Faculty of Pharmacy, Hacettepe University, 06100 Ankara, Turkey; 2 Graduate School of Pharmaceutical Sciences, Kumamoto University, Chuo-ku, Kumamoto 862-0973, Japan; 3 Department of Biology, Science Faculty, Selcuk University, 42130 Konya, Turkey; 4 Department of Food Technology, University Center Koprivnica, University North, 48000 Koprivnica, Croatia

**Keywords:** Alzheimer’s disease, biflavonoids, ginkgetin, neuroprotection, ginkgo

## Abstract

Neurological disorders are becoming more common, and there is an intense search for molecules that can help treat them. Several natural components, especially those from the flavonoid group, have shown promising results. Ginkgetin is the first known biflavonoid, a flavonoid dimer isolated from ginkgo (*Ginkgo biloba* L.). Later, its occurrence was discovered in more than 20 different plant species, most of which are known for their use in traditional medicine. Herein we have summarized the data on the neuroprotective potential of ginkgetin. There is evidence of protection against neuronal damage caused by ischemic strokes, neurotumors, Alzheimer’s disease (AD), and Parkinson’s disease (PD). Beneficial effects in ischemic strokes have been demonstrated in animal studies in which injection of ginkgetin before or after onset of the stoke showed protection from neuronal damage. AD protection has been the most studied to date. Possible mechanisms include inhibition of reactive oxygen species, inhibition of β-secretase, inhibition of Aβ fibril formation, amelioration of inflammation, and antimicrobial activity. Ginkgetin has also shown positive effects on the relief of PD symptoms in animal studies. Most of the available data are from in vitro or in vivo animal studies, where ginkgetin showed promising results, and further clinical studies should be conducted.

## 1. Introduction

Since 1840, human life expectancy has increased at a rate of nearly 2.5 years per decade, and this trend has continued to this day [1]. According to the World Health Organization [2], by 2030, 1 in 6 people in the world will be 60 or older. At that time, the proportion of the population aged 60 and over is estimated to increase from 1 billion in 2020 to 1.4 billion. By 2050, the global population aged 60 and older will double (2.1 billion). Similarly, the number of people aged 80 or older is estimated to triple between 2020 and 2050, reaching 426 million. On a biological level, aging is a complex process in which a variety of molecules and cellular damage accumulate, leading over time to a gradual decline in physical performance and cognitive functions, as well as an increased risk of disease. Older people are more susceptible to various chronic diseases, especially diseases of the central nervous system, such as strokes, epilepsy, Parkinson’s disease (PD), Alzheimer’s disease (AD), neuropathy, and other dementias [3]. Neurological disorders are disorders of the nervous system and can affect the activity and physiology of the brain, spinal cord, and nerves. They occur in 5% to 55% of people who are aged 55 and older and are associated with a high risk of adverse health effects, including mortality, disability, and hospitalization [3]. Therefore, scientists have made considerable efforts to understand the pathophysiology of these disorders and to develop effective prevention methods and therapies. However, the reported compounds/active ingredients, which are mostly synthetic, are not considered to be very reliable and therapeutically effective due to their complexity and off-target problems [4]. On the other hand, several natural products may prove to be viable preventive therapeutics to fill the large gap in the treatment of neurological disorders [4].

One of the groups of natural products associated with neuroprotective properties is the flavonoids [5,6,7], a large and diverse group of specialized plant metabolites characterized by a 15-carbon flavone backbone (C6-C3-C6) with two benzene rings (A and B) linked by a trinuclear pyran ring (C) [8]. Flavonoids can be mainly divided into six groups: flavones, flavonols, flavan-3-ols, flavanonols, flavanones, isoflavones, and anthocyanins. They can be present in free form in plants, but are more often glycosylated, methylated, acetylated, prenylated, or polymerized [9]. The pattern of conjugation, glycosylation, or methylation is responsible for the different chemical and biological properties of these compounds [10]. Flavonoid dimers known as biflavonoids are formed by two linked flavonoid monomers and consist of flavone-flavone, flavane-flavane, flavane-flavone subunits, and in rare cases, dimers of chalcones and isoflavones. Today, nearly 600 different biflavonoids are known to occur in ferns, bryophytes, angiosperms and gymnosperms [11,12]. They are often found in plants used in traditional medicine and are considered responsible factors in the health benefits of these plants [13]. Biflavonoids possess diverse biological activities including therapeutic potential against neurodegenerative diseases [14].

**Figure 1:**
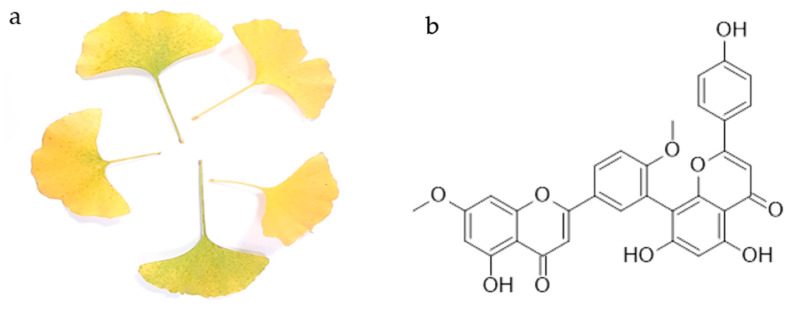
Yellow leaves of *Ginkgo biloba* L. (**a**) and the chemical structure of ginkgetin (**b**).

The first biflavonoid isolated was ginkgetin from the yellow leaves of ginkgo (*Ginkgo biloba* L.) (Figure 1a). Chemically, it is a 7,4′-dimethyl ether derivative of the 3′,8″-dimer of the apigenin, known as amentoflavone. Thus, ginkgetin consists of apigenin and apigenin 7,4′-dimethyl ether.

Ginkgetin is a compound found in ginkgo whose standardized extract (EGb 761) has been used for many years as a supportive therapy and to prevent cognitive impairment [15]. Ginkgo extract can slow the progression of memory loss in AD, usually at a high dose of 240 mg or more per day [16,17], and may have supportive and/or protective effects in the treatment of PD [18]. It is not entirely clear which molecules from the extracts contribute to this activity. Recently, natural products, especially polyphenols, have been intensively studied as potential neuroprotective molecules. One of these molecules is ginkgetin, but as far as we know, there is no review paper summarizing the research to date. Therefore, the review aimed to summarize the data on the potential of ginkgetin in the treatment of neurodegenerative diseases in order to highlight the neuroprotective properties of ginkgetin.

## 2. Ginkgetin

Ginkgetin (Figure 1b) is a flavonoid dimer, a 7,4′-dimethyl ether derivative of the apigenin dimer amentoflavone. It is the first isolated biflavonoid obtained in the form of a yellow powder from the leaves of ginkgo (Figure 1a) and the first biflavonoid whose structure was described. To date, its occurrence has been confirmed in other ginkgo plant parts [13,19], as well as in more than 20 other plant species [20]. The list of plant species in which the presence of ginkgetin was detected is given in Table 1. It should be noted that the presence of ginkgetin in mosses and liverworts has not been yet reported, and in ferns and fern allies it has been reported only in *Sellaginela* sp. It is commonly found in the conifers, cycads and allies group, and in flowering plants. So far, it has not been found in plants commonly used as food, but rather in plants used in traditional medicine.

**Figure 2:**
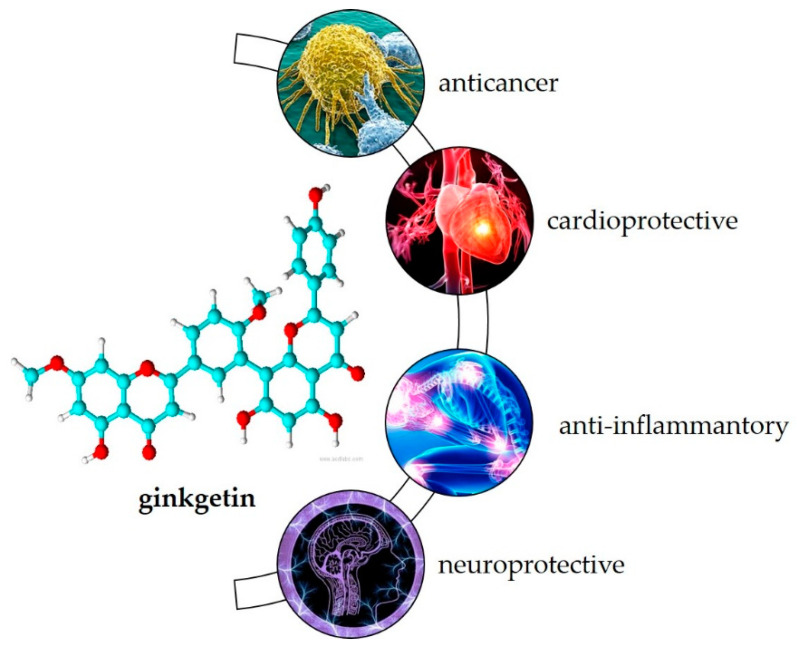
Biological activity of ginkgetin.

Most of the plants listed in Table 1 have been used in the traditional medicine systems of various cultures, suggesting that ginkgetin also may have biological activity. As the first known biflavonoid, its biological activity has been studied over the last 30 years. Research shows its potential in treating various inflammation-related diseases such as cancer, cardiovascular disease, inflammation caused by viruses and bacteria, and neurodegenerative disorders [20] (Figure 2).

Most commonly, its anticancer activity has been studied. Recently, Adnan et al. [20] summarized that ginkgetin combats cancer progression by various mechanisms such as arresting the cell cycle, inducing apoptosis, stimulating autophagy, and targeting many deregulated signaling pathways such as JAK / STAT and MAPKs in the colon, lung, prostate, osteosarcoma, breast, leukemia, cervical, medulloblastoma, ovarian, neck, and kidney cell lines. In animal studies, ginkgetin inhibited tumor growth in xenotransplanted nude mice, down-regulated p-STAT3Tyr705 and survivin in tumor tissues [46] and decreased tumor size and weight without apparent toxicity [47]. Ginkgetin may also enhance the therapeutic effects of cisplatin [48] and 5-fluorouracil [49].

It may also be useful for the treatment of cardiovascular disease. Cell-based studies showed its potential as an inhibitor of TRPV4-mediated proatherogenic processes in macrophages [50]. In addition, ginkgetin showed inhibitory effects on human thrombin, an important serine protease that regulates the blood coagulation cascade and processes of thrombosis [51], and pancreatic lipase, an important target that regulates lipid uptake [52]. Animal studies showed its beneficial effects in preventing adipogenesis [53], local vascular damage associated with atherosclerosis [54], and ischemic reperfusion injury [55].

Due to its anti-inflammatory effects, in vitro studies have shown that ginkgetin can be used in the treatment of inflammation-related diseases such as airway inflammation [56] and diabetic nephropathy [57]. It may also be useful as an antiviral [32], antibacterial [30] and antiparasitic [58] agent and has gained attention in recent years as a target for the treatment of SARS-CoV-2 infection [59,60,61].

In this review, we address in more detail the potential role of gingketin in the treatment of neurodegenerative diseases.

## 3. Neurodegenerative Diseases

**Figure 3:**
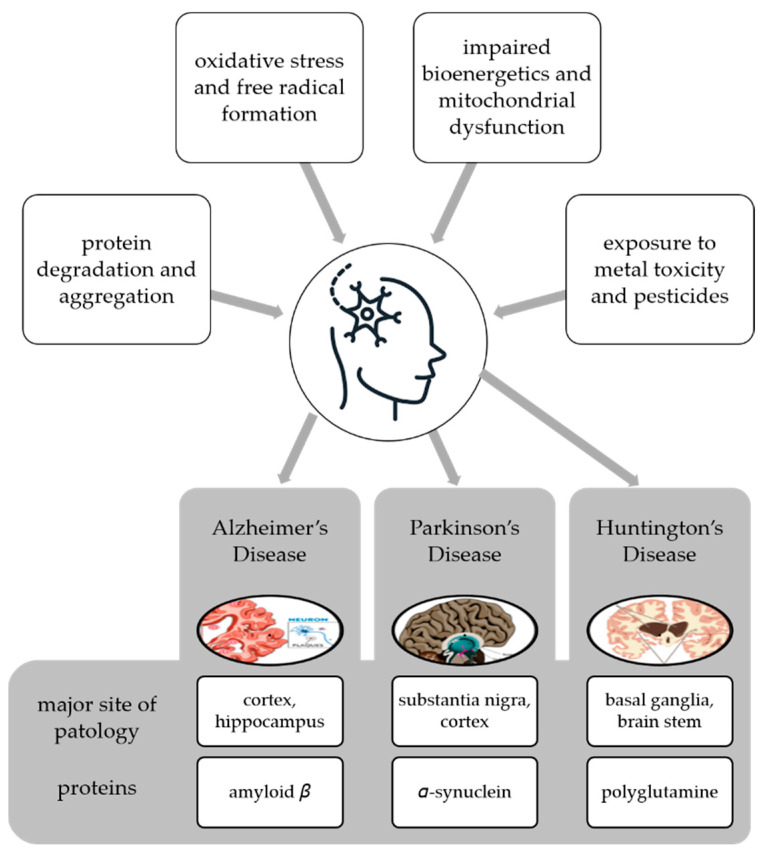
Multifactorial conditions causing neurodegenerative diseases and examples of diseases with the main site of pathology and proteins whose degradation and aggregation cause the pathology.

Neurodegenerative disease is a general term for several diseases mainly characterized by a progressive loss of structure or function of neurons that worsens over time [62]. They can be genetic or caused by a tumor, stroke, toxins, viruses, etc., and affect millions of people around the world. Neurodegenerative disorders are caused by various conditions such as abnormal protein dynamics with defective protein aggregation and degradation and aggregation, impaired bioenergetics and mitochondrial dysfunction, excessive free radical formation leading tooxidative stress, and exposure to environntal toxicants such as heavy metals and pesticides [63] (Figure 3).

Neuroprotection is defined as the ability of a specific molecule to prevent neuronal cell death by interfering with and inhibiting the pathogenetic cascade that leads to cell dysfunction and eventual death [64]. AD and PD are the most common neurodegenerative diseases, but others can cause serious problems for individuals and society, and lead to significant healthcare costs. These diseases are known that these diseases cause irreversible cognitive dysfunctions in individuals. It is therefore extremely important for an effective treatment strategy to slow down the prognosis of neurodegenerative diseases by diagnosing them at the earliest possible stage. AD, PD, Huntington’s disease (HD), and other neurodegenerative disorders share common features at the cellular and subcellular levels, and utilize similar molecular signaling pathways that can lead to inflammation, apoptosisnecroptosis, etc. These diseases are the consequence of misfolding and dysfunctional trafficking of proteins (Figure 3), mitochondrial dysfunction, oxidative stress, and/or environmental factors [63].

AD is a highly complex disorder characterized by severe synaptic losses and neuronal death, especially in regions with cognitive functions such as the cerebral cortex, hippocampus, entorhinal cortex, and ventral striatum [65]. Generally, in an average of 10 years, the stage of mild cognitive impairment passes to the advanced stage of AD, and the patient is lost in a completely helpless state at the end of this period. Due to the long duration of the disease and the fact that it affects the vital structures that determine who we are, it creates a great emotional and financial burden on patients’ relatives and society [66]. Since the pioneering work of Alois Alzheimer in 1907, neuropathologists have identified amyloid plaques and NFTs in the brains of patients in autopsy examinations and stated that these pathologies cause the disease [67]. Amyloid plaques have been found to be extracellular deposits of amyloid-beta (Aβ) found in the brain parenchyma and cerebral blood vessels. The NFTs observed in the cell were found to consist of hyperphosphorylated tau protein associated with microtubules and clustered in helical filaments [68]. Additional pathological data for amyloid plaques and NFTs can be listed as intracellular granulovacuolar degeneration, decrease in the number of synapses, cholinergic cell losses in Meynert’s basal nucleus, and astroglial activation [69]. Studies conducted to understand AD indicate that the disease arises as a result of complex interactions of many genetic, epigenetic, and environmental factors [69,70]. The main histopathological findings observed in the brain parenchyma of the patients have extracellularly located amyloid plaques, neurofibrillary structures consisting of intracellular tau protein clusters, glial activation, and traces of inflammation [71]. Based on these symptoms, many mechanisms have been proposed for the pathogenesis of the disease. The main ones can be listed as the amyloid cascade hypothesis, cholinergic damage hypothesis, neuronal cytoskeleton hypothesis, and oxidative stress hypothesis [72,73]. Other more debatable AD hypotheses are: inflammatory hypothesis, vascular hypothesis, cholesterol hypothesis, metal hypothesis, and cell cycle hypothesis [74].

PD is a progressive neurodegenerative disease that causes involuntary or uncontrollable movements such as tremors, stiffness, balance and coordination problems [75]. Symptoms usually begin insidiously and worsen over time. Degeneration of neurons in the compacta part of substantia nigra and the presence of Lewy bodies in its cytoplasm are the classic pathological findings of the disease [76]. Over the years of PD progression, a picture of dementia can develop that can be severe and debilitating, overshadowing the movement disorder of the disease. Dementia is defined as the presence of impairments in more than one cognitive domain, such as attention, memory, language, executive functioning, practice, and visuo-spatial functioning [77]. These losses reflect a marked decline from previous levels, and this decline is severe enough to interfere with daily, occupational, and social life. PD dementia is a mild or moderate dementia that begins insidiously, progresses slowly, affects some areas of cognitive function, especially executive function, and often develops psychosis during its course. The mechanisms underlying the pathogenesis of PD are still unclear, but there are several proposed mechanisms, such as those related to mitochondrial dysfunction, oxidative stress, ubiquitin-proteosome system, neuroinflammation, excitotoxicity, iron ion accumulation, and genetic issues [78].

HD is an autosomal dominant genetic disorder mainly characterized by progressive motor dysfunction, cognitive decline, and behavioral symptoms [79]. Amyotrophic lateral sclerosis (ALS) is a fatal late-onset neurodegenerative disorder that is characterized by a progressive loss of motor neurons of the CNS leading to muscle weakness, wasting, and spasticity. Patients develop progressive muscle weakness along with fasciculation and hyperreflexia. Mild cognitive deficits and frontotemporal dementia (FTD) are common. FTD is characterized by progressive deficits in executive function, behavior, and language.

Neurodegenerative diseases also share some common pathological features such as the accumulation of characteristic proteins in insoluble aggregates within and/or between neurons and the loss of synapses and death of neurons [80]. These proteins include β-amyloid (Aβ) of senile plaques and tau of neurofibrillary tangles (NFTs) in AD, α-synuclein (α-syn) of Lewy bodies (LBs) and Lewy neurites in PD, polyglutamine (PolyQ)-rich huntingtin inclusions in HD, TDP-43 aggregates in ALS, and TDP-43 aggregates and tau in FTD (Figure 3). In line with the above-mentioned explanations, neurodegenerative diseases occur with the folding and proteasomal disorders of certain proteins due to environmental or genetic reasons, followed by active glial cells secreting various mediators, including proinflammatory cytokines [80]. This whole process repeats each other in a vicious circle, causing apoptosis and developing neuroinflammation. This mechanism is the underlying cause of all diseases. Therefore, it is necessary to develop treatment strategies against neuroinflammation. These may include the use of natural sources or their secondary metabolites with anti-inflammatory effects.

## 4. Ginkgetin for the Treatment of Neurodegenerative Diseases

### 4.1. Oxidative Stress Mediation

**Figure 4:**
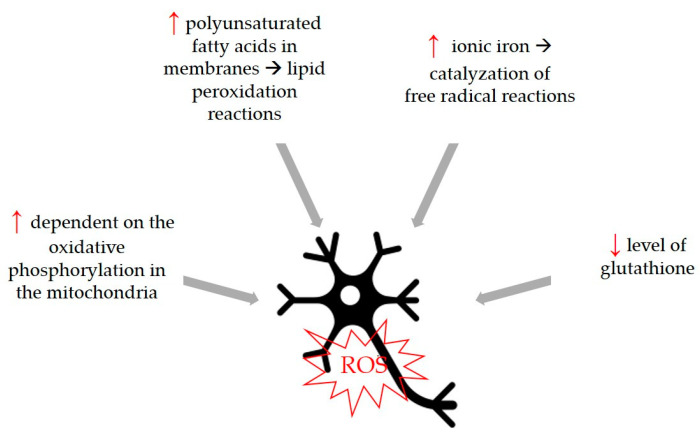
Neurons are more susceptible to free radical damage because of differences in some parameters and biological functions. Higher parameters are marked with **↑**, lower parameters with **↓**.

Oxidative stress is a common trigger that can be associated with the development of neurodegenerative diseases, so compounds with antioxidant activity can be considered beneficial for the development of these diseases. Although the physiological cause of aging is not fully known, the free radical theory states that increasing oxidative stress of aging and aging-related diseases plays a fundamental role in this process by causing cellular degeneration. The increase in the number of free radicals observed in age-related neurodegenerative diseases and the fact that neurons are more sensitive to this damage have both been determined to be important characteristics. Therefore, it is thought that free radical production has an important role in the development and progression of neurological diseases [81]. Neurons are more susceptible to free radical damage [82] for certain reasons shown in Figure 4.

Flavonoids are widely recognized as a molecule with good antioxidant activity and beneficial effects in the treatment of neurological disorders [5,6,7,8,9,10,11,12,13,14,15,16,17,18,19,20,21,22,23,24,25,26,27,28,29,30,31,32,33,34,35,36,37,38,39,40,41,42,43,44,45,46,47,48,49,50,51,52,53,54,55,56,57,58,59,60,61,62,63,64,65,66,67,68,69,70,71,72,73,74,75,76,77,78,79,80,81,82,83]. Li et al. [84] measured the antioxidant activity of ginkgo leaves from plants grown in different locations and reported that ginkgetin content in the leaves resulted in stronger antioxidant activity and concluded that ginkgetin together with isoginkgetin were the two most active constituents with a strong relationship with antioxidant activity. Several cell-based studies have shown that ginkgetin plays a role in oxidative stress and that ginkgetin can protect fibroblasts from UVB-induced cytotoxicity [85], alleviate oxidative stress induced by H/R injury [86], inhibited NO production from lipopolysaccharide (LPS)-induced RAW 264.7 cells [87] and reduced oxidative stress caused by hyperglycemia [57]. However, reports about the antioxidant activity of ginkgetin are contradictory. Bedir et al. [40] who compared the antioxidant activity of 29 compounds isolated from *G. biloba* reported that ginkgetin was the least potent antioxidant after amentoflavone, showing only 19% inhibition at a concentration of 62.5 µg/mL. In the same study, monomeric biflavonoids had an IC_50_ of less than 10 µg/mL, clearly indicating that ginkgetin itself is not a molecule with antioxidant potential. Kang et al. [14] tested protective effect of biflavonoids on H_2_O_2_-induced cell death in SH-SY5Y (triple subcloned cell line derived from SK-N-SH neuroblastoma) and showed that all biflavonoids tested, including ginkgetin, significantly reduced H_2_O_2_-induced cell death. Furthermore, they tested the antioxidant activity of using the well- known 1,1-diphenyl-2-picrylhydrazyl (DPPH) assay, and none of the nine biflavones showed radical scavenging activity at concentrations up to 100 µM. These results suggest that the neuroprotection of biflavonoids may be mediated by direct blockade of cell death cascades rather than by their antioxidant activity. Therefore, they investigated the neuroprotective effects of biflavonoids against the cytotoxic insult induced by staurosporine. Staurosporine is known to mediate apoptosis via the caspase-dependent mitochondrial pathway. Ginkgetin significantly reduced cell death induced by staurosporine at a concentration of 10 µM. They therefore suggest that neuroprotection by biflavonoids is mediated in part, if not completely, by direct blockade of the signaling events that lead to apoptosis during cellular stress. Jeong et al. [31] investigated the neuroprotective effects of four biflavonoids, including ginkgetin, using mouse HT22 hippocampal cells, a model system for studying glutamate-induced oxidative stress. They reported that ginkgetin can protect HT22 neuronal cells from glutamate-induced oxidative damage by preserving the activities of antioxidant enzymes and/or inhibiting ERK1/2 activation.

This example shows that commonly used methods for determining antioxidant activity, such as DPPH, are not ideal for predicting the ability of compounds to reduce oxidative stress at the cellular level and even more so at the tissue or organism level.

### 4.2. Protection against Neuronal Injury Caused by Ischemic Stroke

Ample evidence has supported the role of neuroinflammation in the development of neurological disorders. Inflammatory components such as astrocytes, microglia, the complement system, and cytokines have been associated with neuroinflammation in the CNS. In particular, inflammatory cytokines have been found to play a central role in neuroinflammation pathway as a large numberof studies have reported abnormally elevated levels of interleukin-1β (IL-1β) and tumor necrosis factor (TNF) in AD and PD patients (reviewd by [88]).

**Figure 5:**
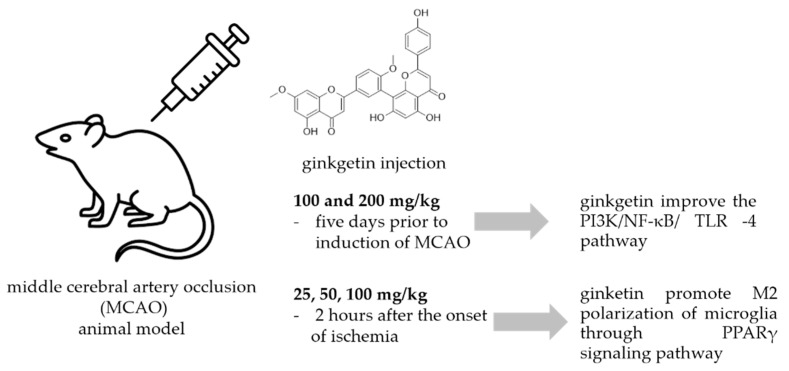
Ginkgetin protection against neuronal injury caused by ischemic stroke.

A common method to study the antineuroinflammatory potential of certain molecules is to use animal models exposed to neuronal injury caused by ischemic stroke. Most ischemic strokes occur in the middle cerebral artery territory, so many of the animal models of stroke that have been developed have focused on this artery. In the intraluminal monofilament model of middle cerebral artery occlusion (MCAO), a surgical suture is inserted into the external carotid artery and advanced into the internal carotid artery (ICA) until the tip occludes the origin of the MCA, resulting in interruption of blood flow and subsequent cerebral infarction in the MCA area [89]. This model has been used also for the study of the effects of ginkgetin (Figure 5.).

In a study by Xu et al. [90] animals received ginkgetin at concentrations of 100 and 200 mg/kg i.p. five days prior to induction of MCAO, and they investigated the effect of ginkgetin against stroke. Treatment with ginkgetin attenuated the increased neurological score and decreased the water content in the brain. Ginkgetin-treated rats showed that the levels of pro-inflammatory cytokines NF-κB, IL-1β, and TNF-α were significantly decreased in brain tissue. The authors concluded that ginkgetin aglycone improved the PI3K/NF-κB/ TLR-4 inflammatory pathway. Tian et al. [91] used a transient MCAO procedure to establish the cerebral ischemia/reperfusion model (IR) in rats. Ginkgetin was injected at doses of 25, 50, and 100 mg/kg 2 hours after the onset of ischemia and its administration markedly reduced the volume of cerebral infarction and neurologic deficits. It also reduced the number of apoptotic cells, decreased the amount of cleaved caspase-3 and Bax, and increased the amount of Bcl-2 in rats exposed to IR injury in a dose-dependent manner. In addition, high-dose ginkgetin treatment (100 mg/kg) significantly increased the phosphorylations of Akt and mTOR. Blocking PI3K by LY294002 significantly decreased the antiapoptotic effect and reduced both Akt and mTOR phosphorylation levels. According to the authors, ginkgetin counteracts cerebral IR-induced injury by inhibiting apoptosis in rats, and this effect was attenuated by activation of the PI3K/Akt/mTOR pathway. The same experiment procedure and the same ginkgetin concentration was used by Pan et al. [92] who reported that ginkgetin attenuated I/R-induced autophagy activation, pyramidal neuron death in cerebral I/R, and reduced I/R-induced upregulation of p53. They concluded that ginkgetin can attenuate cerebral ischemia/reperfusion-induced autophagy and apoptosis by inhibiting the NF-κB/p53 pathway. Some other researchers [93] used oxygen glucose deprivation (OGD) cellular and MCAO animal models to study neuroprotective activity of ginkgetin reported that ginkgetin treatment converted microglia from M1 type to M2 type and inhibited neuroinflammation. Detailed study of the neuroprotective mechanism suggested that ginkgetin can inhibit neuroinflammation by promoting M2 polarization of microglia through PPARγ signaling pathway thus promoting recovery of neurological functions in an ischemic stroke.

### 4.3. Activity against Neurotumors

Different *in vitro* and *in vivo* studies showed that ginkgetin combats cancer progression by arresting the cell cycle, inducing *via* apoptosis, stimulating autophagy, and targeting many deregulated signaling pathways such as JAK/STAT and MAPKs (reviewed by Adnan et al. [20]). Ginkgetin was also studied as a potential agent for the treatment of neurotumores by Ye et. al. [26], who investigated the potential of natural products in the treatment of medulloblastoma (MB), a form of malignant brain tumor that occurs predominantly in infants and children and in which approximately 25% is due to upregulation of the canonical Wnt pathway, with mutations mainly in CTNNB1. They screened for antagonists of Wnt signaling from 600 natural compounds and identified ginkgetin as a potential molecule that showed marked cytotoxicity. Ginkgetin efficiently induced G2/M phase arrest in Daoy cells, reduced the expression of Wnt target genes, including Axin2, CyclinD1, and Survivin in MB cells, and decreased the phosphorylation level of β-catenin. They concluded that ginkgetin is a novel inhibitor of Wnt signaling and, as such, warrants further exploration as a promising candidate against medulloblastoma.

### 4.4. Protective Effect against Alzheimers’ Disease

AD is caused by multiple mechanisms such as excessive accumulation of extracellular amyloid-beta 42 (Aβ42) plaques, intracellular hyperphosphorylated tau neurofibril tangles in the brain, oxidative stress due to mitochondrial dysfunction, and/or genetic as well as environmental factors [94]. Aggregation and accumulation of amyloid-β plaques and tau proteins in the brain are central features in the pathophysiology of AD and are therefore the focus of most research investigating potential therapeutics for this neurodegenerative disease [95]. Kang et al. [14] investigated whether biflavones showed protective effects against Aβ-induced cytotoxicity using SH-SY5Y (triple subcloned cell line derived from SK-N-SH neuroblastoma) cells and found that ginkgetin showed protective effects at 2 µM, with an inhibition percentage of 43.6%. In the same study they tested protective effects against neuronal cell death induced by a DNA-damaging agent, etoposide, but gingetin did not show a protective effect.

Amyloid-β-42 (Aβ42) is proteolytic derivative of the large transmembrane protein amyloid precursor protein (APP) and it plays an early and important role in all cases of AD [96]. Thus, blocking Aβ42 production by specific inhibition of key proteases required for Aβ42 formation is a major focus of AD therapy research. β-Secretase, the aspartic protease that generates the N-terminus of Aβ42, has become a major target and researchers are focused on discovering its inhibitors [96]. Sasaki et al. [97] examined the activity of twenty-one bioflavonoids against β-secretase and ginkgetin showed a significant inhibitory effect with an IC_50_ value of 4.18 µM. The authors indicated that the importance of the position of hydroxyl groups in two apigenin molecules for the inhibition of β-secretase and the presence of hydroxyl groups in the C3′ and C8″ position might enhance the inhibitory effects. Ullah et al. [98] reviewed β-secretase inhibitors from plant sources and, among them, ginkgetin was a significant inhibitor with a low IC_50_ value. In an in silico study performed by Grewal et al. [99], ginkgetin showed a good binding potential on N-methyl-D-aspartate glutamate receptor (NMDA) and beta secretase-1 (BACE-1), and was suggested as a neuroprotective agent. In another study conducted by Choi et al. [100], eight amentoflavone-like bioflavonoids were tested to inhibit amyloid-beta fibrillation and to disaggregate amyloid-beta fibrils. In the study, the IC_50_ value of ginkgetin was 4.92 µM in the inhibition of Aβ fibrils assay. In the same study, ginkgetin exhibited a disaggregation effect on Aβ fibrils with the IC_50_ value of 6.81 µM.

Zeng et al. [101] studied ginkgetin therapeutic potential against AD using a transgenic mouse model of AD, PS1dE9/APPS mice. Prior to the onset of AD-type neuropathology, mice were randomly assigned to four diet groups: ginkgetin group, curcumin group, normal diet group, with wild-type littermates used as a control group. All animals were fed with the above diets for 9 months. The mean daily food consumption of the mice was 0.08– 0.12 g/g body weight and the corresponding daily ginkgetin and curcumin were about 200 and 80 mg/kg/day based on a previous report indicating lack of toxicity. The equivalent consumption in a 60 kg human is about 0.91 g/day for ginkgetin and 0.35 g/day for curcuminas. In their experiments they showed that ginkgetin effectively reduced the Aβ levels in the brain and blood, decreased cerebral microhemorrhage, lowered astrogliosis, and ameliorated inflammation in APP/PS1 transgenic model, which indicates in vivo therapeutic potential of ginkgetin against AD. However, as the authors stated, pathophysiology mechanisms of Aβ clearance need further research.

**Figure 6:**
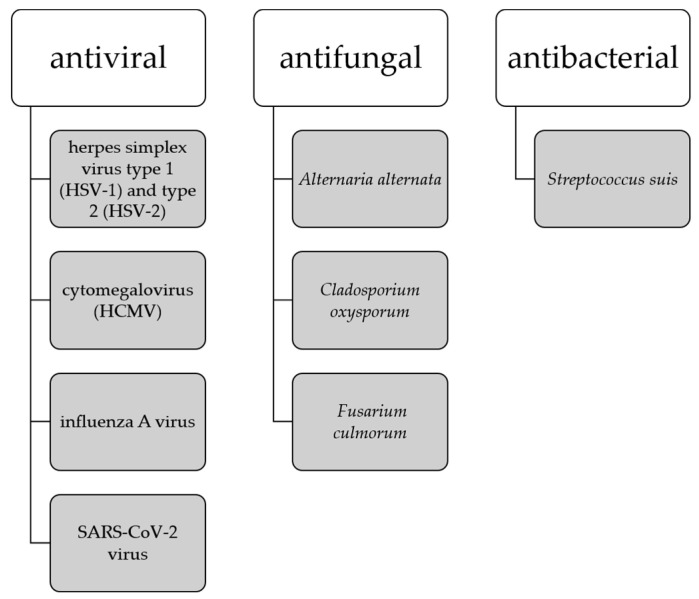
Antimicrobial activity of ginkgetin.

It has been noted that the development of amyloid-β plaques occurs about 10–20 years before the manifestation of AD symptoms, thus the earlier interventions are necessary to address presymptomatic AD. Studies suggesting that amyloid-β peptides may play a role in innate immunity as antimicrobial peptides indicate that the buildup of amyloid-β plaques may be a response to the presence of viruses and bacteria [94]. This has led to the establishment of the antimicrobial hypothesis for AD and the use of antimicrobial and antiviral drugs as potential therapeutics targeting the root cause of AD. Biflavonoids are in the focus of the sciences as a potent antimicrobial, expecially antiviral agents [13,94] where ginkgetin stands out as a compound with antiviral capabilities against herpes simplex virus type 1 (HSV-1) and type 2 (HSV-2) [25], cytomegalovirus (HCMV) [25], influenza A virus [32], and SARS-CoV-2 virus [59,60,102]. Ginkgetin also shows antifungal activity against *Alternaria alternate, Cladosporium oxysporum*, and *Fusarium culmorum* [35], and antibacterial activity against *Streptococcus suis* [103] (Figure 6).

At this point, researchers have not linked any specific bacterium or virus alone to the development of AD. Thus, a number of viruses and bacteria may be involved in the progression of neurodegenerative diseases independently or simultaneously with other pathogens. Given the good antimicrobial, especially antiviral, activity of ginkgetin, its potential role in mechanisms related to the antimicrobial hypothesis for AD is worthy of future research.

**Figure 7:**
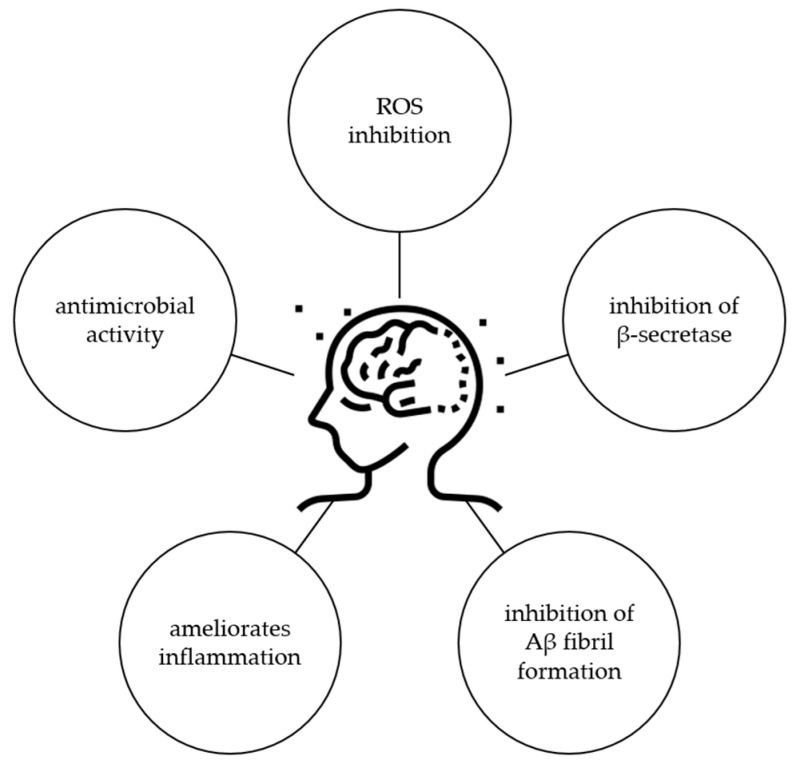
Biological activity of ginkgetin related to protection against AD.

Considering all these results, it is obvious that ginkgetin has potential for the treatment of AD, but further studies should be performed to confirm this activity, especially in a clinical trial. Possible mechanisms of ginkgetin related to the protection of AD are shown in Figure 7.

### 4.5. Protective Effect against Parkinson’ Disease

PD is reported to be the second most common neurodegenerative disorder after AD [104]. Therefore, great efforts have been made to search for new molecules that could be effective in the treatment of PD. Although there are several in silico and in vitro models for finding new active molecules, our literature search did not yield any results related to ginkgetin. The animal model commonly used is a model where PD is induced by 1-methyl-4-phenyl-1,2,3,6-tetrahydropyridine (MPTP), which is the gold standard for researchers in order to induce all aspects of PD hallmarks in animal model of the disease [104]. Wang et al. [105] investigated the neuroprotective ability of ginkgetin in vivo in a model of PD induced by MPTP. Animals received ginkgetin (80 mg/100 g body weight) via the stomach for 5 days and then were injected intraperitoneally with MPTP (20 mg/kg) once daily for 5 days. The authors showed that ginkgetin significantly improved sensorimotor coordination in a mouse model PD by dramatically inhibiting the decline in tyrosine hydroxylase expression in the substantia nigra and superoxide dismutase activity in the striatum. They reported that ginkgetin can strongly chelate iron ions, thereby inhibiting the increase in intracellular labile iron pool by downregulating L-ferritin and upregulating transferrin receptor 1, suggesting that the neuroprotective mechanism of ginkgetin against neurological damage induced by MPTP is via the regulation of iron homeostasis. In another animal study [106], mice were treated with 1-methyl-4-phenyl-1,2,3,6-tetrahydropyridine (MPTP) (25 mg/kg) and probenecid (250 mg/kg) for five consecutive days to induce PD. Ginkgetin (5, 10, 20 mg/kg) and bromocriptine (10 mg/kg), which is used to treat PD, were administered orally to PD mice for 26 days, including a five-day pretreatment period. The authors reported that in MPTP-induced PD mice, movements and muscle functions improved with ginkgetin. The number of tyrosine hydroxylase-positive cells was reduced and later recovered without degeneration. The level of glial fibrillary acidic protein (GFAP) decreased, while the level of brain-derived neurotrophic factor (BDNF) increased significantly after treatment with ginkgetin. In summary, the authors concluded that ginkgetin effectively protects dopaminergic neurons by reducing oxidative damage, activating microglia, and increasing neurotrophic potential, indicating that it is a potential candidate for the treatment of PD.

## 5. Conclusions

Ginkgetin is the first biflavonoid isolated from ginkgo, after which it was named. All biflavonoids belong to the flavonoid group, well-studied specialized metabolites from plants, but they are much less studied compared to monomeric flavonoids. In this review, we have summarized the available data on the neuroprotective potential of ginkgetin. The available data are in vitro studies or in vivo animal studies, and as far as we know, there have been no clinical studies performed as yet. There is evidence of protection against neuronal damage caused by ischemic stroke, neurotumors, AD, and PD, but further studies and clinical trials should explain the mechanisms of action and the effective and safe concentration of ginkgetin for clinical use. The focus of future research should be primarily on the potential to cross the blood-brain barrier, as there is currently a lack of information in this regard.

## Figures and Tables

**Table 1:** List of plant species with associated division in which the presence of ginkgetin has been reported.

Division	Species
**Thallophyta** Unicellular to large algae, fungi, lichens	data not available
**Bryophyta** Mosses and liverwords	data not available
**Pteridophyta** Ferns and fern allies	*Selaginella doederleinii* [21] *Selaginella moellendorffii* [22] *Selaginella sinensis* [23]
**Gymnosperms** Conifers, cycads and allies	*Araucaria angustifolia* [24] *Cephalotaxus drupacea* [25] *Cephalotaxus fortunei* var. *alpina* [26] *Cephalotaxus harringtonia* var. *harringtonia* [27] *Cephalotaxus koreana* [28] *Cephalotaxus sinensis* [29] *Cycas media* [30] *Chamaecyparis obtusa* [31] *Ginkgo biloba* [32,33,34] *Metasequoia glyptostroboides* [35] *Taxus baccata* [35] *Taxus chinesis* [36] *Taxus cuspidata* [37] *Taxus mairei* [38] *Taxus media* [38] *Torreya nucifera* [39]
**Angiosperms** Flowering plants	*Capparis spinosa* [40] *Celaenodendron mexicanum* [41] *Cyperus rotundus* [42] *Elateriospermum tapos* [43] *Gaultheria yunnanensis* [44] *Houttuynia cordata* [45]

## Data Availability

Not applicable.
